# Reliability of isometric and isokinetic trunk flexor strength using a functional electromechanical dynamometer

**DOI:** 10.7717/peerj.7883

**Published:** 2019-10-18

**Authors:** Angela Rodriguez-Perea, Luis J. Chirosa Ríos, Dario Martinez-Garcia, David Ulloa-Díaz, Francisco Guede Rojas, Daniel Jerez-Mayorga, Ignacio J. Chirosa Rios

**Affiliations:** 1 Department of Physical Education and Sport, Faculty of Sport Sciences, University of Granada, Granada, Granada, Spain; 2 Department of Sports Sciences and Physical Conditioning, Faculty of Education, CIEDE, Catholic University of Most Holy Conception, Concepcion, Concepcion, Chile; 3 Faculty of Rehabilitation Sciences, Universidad Andres Bello, Concepcion, Chile; 4 Faculty of Rehabilitation Sciences, Universidad Andres Bello, Santiago, Chile

**Keywords:** Core, Resistance, Reproducibility, Test, Isokinetic, Isometric

## Abstract

**Aim:**

To determine the absolute and relative reliability of functional trunk tests, using a functional electromechanical dynamometer to evaluate the isokinetic strength of trunk flexors and to determine the most reliable assessment condition, in order to compare the absolute and relative reliability of mean force and peak force of trunk flexors and to determine which isokinetic condition of evaluation is best related to the maximum isometric.

**Methods:**

Test-retest of thirty-seven physically active male student volunteers who performed the different protocols, isometric contraction and the combination of three velocities (V_1_ = 015 m s^−1^ , V_2_ = 0.30 m  s^−1^, V_3_ = 0.45 m s^−1^) and two range of movement (R_1_ = 25% cm ; R_2_ = 50% cm) protocols.

**Results:**

All protocols to evaluate trunk flexors showed an absolute reliability provided a stable repeatability for isometric and dynamic protocols with a coefficient of variation (CV) being below 10% and a high or very high relative reliability (0.69 < intraclass correlation coefficient [ICC] > 0.86). The more reliable strength manifestation (CV = 6.82%) to evaluate the concentric contraction of trunk flexors was mean force, with 0.15 m  s^−1^ and short range of movement (V_1_R_1_) condition. The most reliable strength manifestation to evaluate the eccentric contraction of trunk flexors was peak force, with 0.15 m  s^−1^ and a large range of movement (V_1_R_2;_ CV = 5.07%), and the most reliable way to evaluate isometric trunk flexors was by peak force (CV = 7.72%). The mean force of eccentric trunk flexor strength with 0.45 m  s^−1^ and short range of movement (V_3_R_1_) condition (r = 0.73) was best related to the maximum isometric contraction.

**Conclusion:**

Functional electromechanical dynamometry is a reliable evaluation system for assessment of trunk flexor strength.

## Introduction

Trunk flexion is present in daily activities, such as walking or sit-to-stand (Roldán-Jiménez, Bennett & Cuesta-Vargas, 2015; Silva et al., 2015; Moon & Kim, 2017) , and in different sports performance actions, such as overhead throwing (Van den Tillaar & Ettema, 2009; Liu, Leigh & Yu, 2010; Wagner et al., 2012; Wagner et al., 2014; Palmer et al., 2015) or hitting a ball (Lindsay, Horton & Paley, 2002; Chang et al., 2016). Studies have demonstrated the importance of trunk strength for preventing injuries in the spine (Hutten & Hermens, 1997; Mueller et al., 2012; Rossi et al., 2017) and knee (Araujo, Cohen & Hayes, 2015; Cronström et al., 2016), such as the low back pain and anterior cruciate ligament injuries that frequently occur in sports and the workplace (Leboeuf-Yde et al., 2009; Sanders et al., 2016; Kozak, Freitag & Nienhaus, 2017).

Due to the importance of trunk strength, clinicians and coaches must know whether changes in strength over time reflect a real gain or loss, or are the result of the measurement error (Atkinson & Nevill, 1998). Therefore, the validity and reliability of data are important when assessing strength. The validity of data concern to which an individual’s test performance reflects true performance and the reliability measures in tests and retests concern the repeatability of the data observed in a sample (Hopkins, 2000; Hopkins, Schabort & Hawley, 2001). In sports science, it is a requirement to have relative (intraclass correlation coefficient (ICC)) and absolute reliability (standard error of measurement (SEM) or coefficient of variation (CV)) of data. Relative reliability indicates how similar the rank orders of the participants in the test are to the retest (Weir, 2005). The main problem with relative reliability is that it depends on the variability of the sample. However, absolute reliability is related to the consistency of individual scores (Hopkins, 2000; Hopkins et al., 2009).

Until now, the evaluation of trunk strength has been carried out by way of medicine-ball throws (Glenn et al., 2015), handheld dynamometers (Cowley et al., 2009; Paalanne et al., 2009; Newman, Pollock & Hunt, 2012) or isokinetic devices (Dervisevic, Hadzic & Burger, 2007; Mcintire et al., 2007; Roth et al., 2017). In these isokinetic devices, there is no evaluation protocol to know at what velocity and at what range of movement the evaluation should be performed (Dvir & Müller, 2019), even though there have been attempts (Dvir & Keating, 2001). Still, there are indications that low-velocity movements are more reliable for measuring trunk strength (Guilhem et al., 2014; Roth et al., 2017). Different studies that have analyzed the reliability of test using peak force (Roth et al., 2017; De Blaiser et al., 2018; Juan-Recio et al., 2018) but it has not been shown which strength manifestation (peak force or mean force) is more reliable for assessing trunk strength.

Functional electromechanical dynamometry (FEMD) is a new technology that allows us to evaluate and train strength in human beings. Its provides a quantified measurement of strength and its ease of use and low cost. Unlike other isokinetic devices, this device (DynaSystem, Model Research, Granada, Spain) generates linear isokinetic speeds between other dynamic modes (tonic, kinetic, elastic, inertial, conical) to static (isometric, vibratory) allowing to evaluate and train through resistance/velocity constant and variable (Campos Jara et al., 2014). Furthermore, it has been described as a valid and reliable measurement method for evaluating upper and lower extremity muscle strength (Cerda Vega et al., 2018; Chamorro et al., 2018). This technology has been used to study the isometric strength of the shoulder rotators and hip abductor and has obtained high reliability values (ICC > 0.94; CV < 10%) (Cerda Vega et al., 2018; Chamorro et al., 2018). However, trunk strength has yet to be studied with this device.

Therefore, the main purposes of this study were (I) to determine the absolute and relative reliability of trunk tests with a FEMD (Dynasystem, Symotech) in the evaluation of the isokinetic and isometric strength of trunk flexors, and to determine the most reliable assessment condition, (II) to compare the absolute and relative reliability of mean force and peak force of trunk flexors, and (III) to determine which isokinetic condition of evaluation is best related to the maximum isometric contraction. We hypothesized that (I) low velocities and short range of movement would be more reliable than high velocities and large range of movement. Additionally, we hypothesized that (II) mean force would be a more reliable variable than peak force in trunk flexor evaluation and that (III) slow velocities are best related to isometric evaluation. The results are expected to provide new information regarding trunk strength evaluation protocols using FEMD.

## Methods

### Participants

Thirty-seven physically active male student volunteers (age 21.4 ± 2.1 years, body mass 69.2 ± 6.9 kg, height 1.7 ± 0.1 m and body mass index (BMI) 23.0 ± 1.6 kg/m^2^; data are presented as mean ± standard deviation (SD)) without any experience in isokinetic or dynamometers devices were recruited from the university community (Table 1). Participants were eligible for the study if they were: (I) free of low back pain, with a maximum of 20% in the Oswestry Low Back Pain Disability questionnaire; (II) free of musculoskeletal injury; (III) not practicing specific trunk strength training; and IV) had a maximum BMI of 25 kg/m^2^. All participants were informed regarding nature, aims and risks associated with the experimental procedure before they gave their written consent to participate. The study protocol was approved by the Institutional Review Board of the University of Granada (n° 350/CEIH/2017), and was conducted in accordance with the Helsinki Declaration.

**Table 1:** Characteristic of participants.

	Age (years)	Body mass (Kg)	Height (m)	BMI (Kg/m^2^)	OLBPD (%)	ROM 100% (cm)	ROM 25% (cm)	ROM 50% (cm)
Mean	21.4 (2.1)	69.2 (6.9)	1.7 (0.1)	23.0 (1.6)	3.1 (3.9)	51.4 (3.3)	13.0 (0.9)	25.8 (1.6)
Minimum	19.0	59.1	1.6	19.4	0.0	45.2	11.0	23.0
Maximum	27.0	81.9	1.9	25.0	16.0	59.4	15.0	30.0

**Notes.**

The data are presented mean (SD).

ROM range of movement BMI body mass index OLBPD oswestry low back pain disability

### Study design

A repeated-measurement design was used to evaluate trunk flexor strength with different protocols. All test sessions were developed in the ‘controlled natural movement’ laboratory of the University of the Most Holy Conception (Chile). After two familiarisation sessions, participants attended to the laboratory on two separate days (at least 48 h apart) during two weeks. On each testing day, participants completed different condition of velocity (V) and range of movement (R) protocols. Participants were asked to maintain their physical activity level during the two weeks of the study. All evaluations were conducted by the same evaluator with an experience with the device for more than three years, at the same time of the day (± 1 h) for each participant and under similar environmental conditions (∼21 °C and ∼60% humidity). The order of the velocities and range of movements was randomly established. This order was carried out in the two testing sessions.

#### Instruments

**Figure 1:**
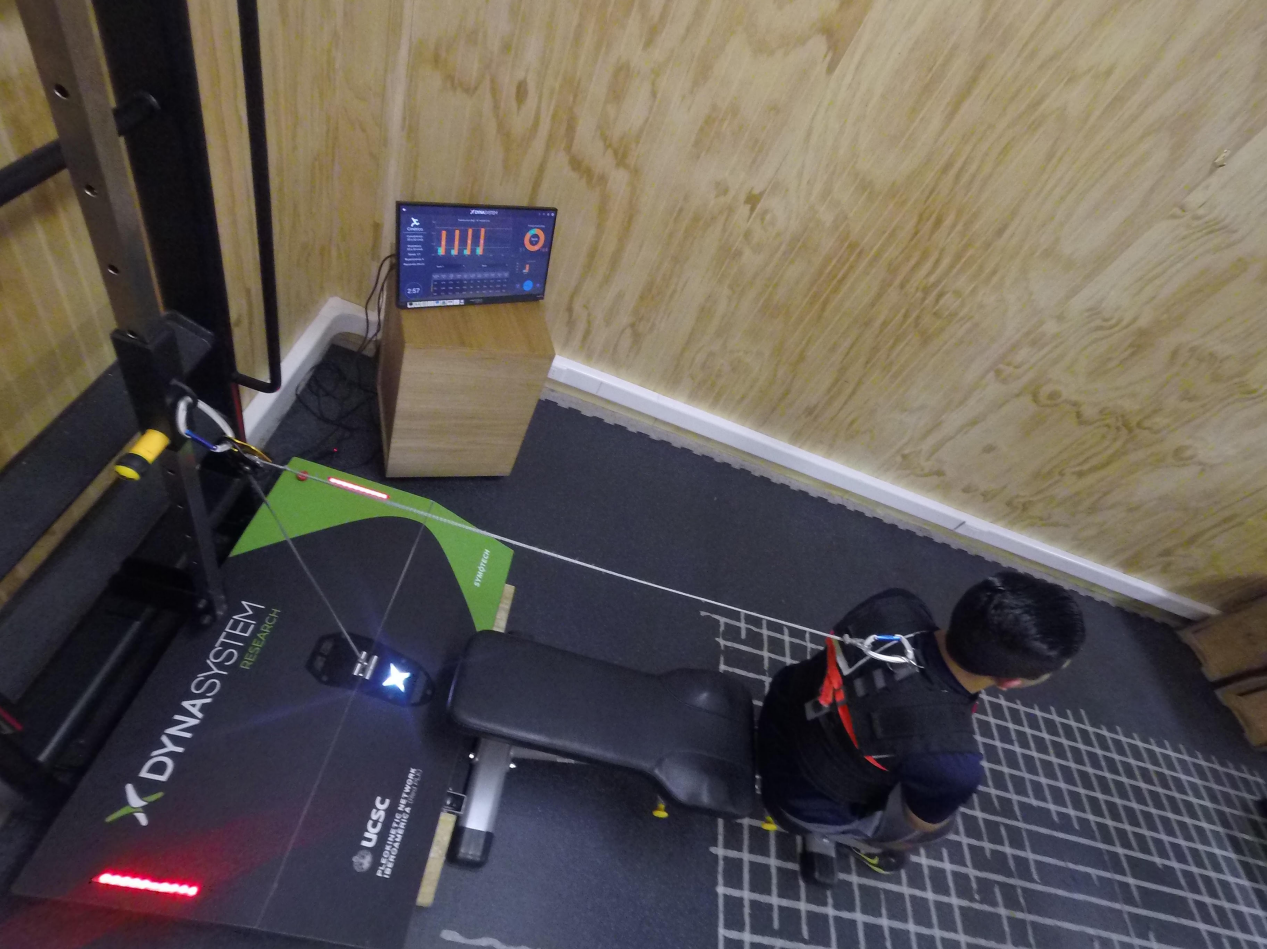
DynaSystem research functional dynamometer.

Isometric and isokinetic strength were evaluated with a DynaSystem Research Functional Dynamometer (SYMOTECH, Granada, Spain) with a precision of three mm for displacement, 100 g for a sensed load, a sampling frequency of 1,000 Hz and a range of velocities between 0.05 m s^−1^ to 2.80 m s^−1^, coupled with a standard bench, a pulley system and a subjection system (Fig. 1).

#### Range of movement

**Figure 2:**
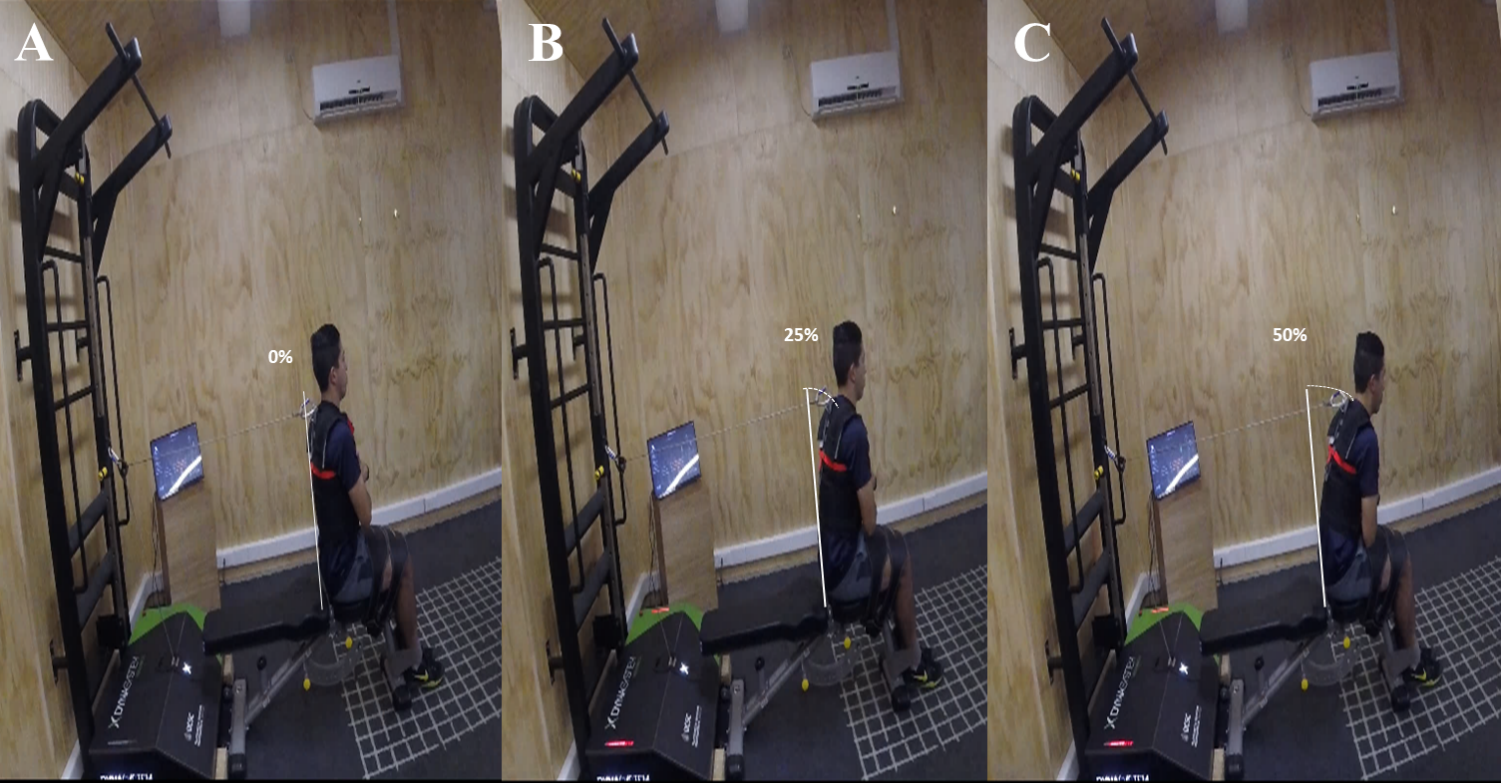
Participant performing a maximum effort of trunk flexion in the functional electromechanical dynamometer with different ROM (A, initial position; B, 25% cm ; C, 50% cm).

The distance between the greater trochanter and the acromion was measured manually to establish the range of movement. Measurements were made by applying anthropometric measurement protocol based on the internationally validated recommendation (Stewart, Marfell-Jones & International Society for Advancement of Kinanthropometry, 2011), using a SECA^®^ brand measuring tape. That distance accounted for 100% of the range of movement. The 25% (R_1_) and 50% (R_2_) of that distance were calculated to establish the range of movement during the execution of the test (Fig. 2). These ROMs are set in the exercise configuration screen on the device prior to the execution of the test and this corresponds to the course of the rope measured in centimeters.

#### Position

The participants were positioned on a flat bench with their feet resting on the floor. Participants were then stabilized at the test position (sitting) using straps. Sliding forward on the bench was avoided with the use of appropriate belts that pushed the pelvis and the legs down and back, but were not uncomfortable for the participants. Because the aim of the study focuses on trunk flexor strength, the seated posture helps to isolate these muscles better, reducing the action of the iliopsoas muscle in a closed kinetic chain. In addition, the sitting posture provides greater stability when fixing the pelvis. The seated knee flexion position reduces hamstring tension, favoring the lumbo-pelvic kinematics (anterior tilting during trunk flexion) and reducing biomechanical stress together with the consequent risk of lumbar pain (Jandre Reis & Ribero Macedo, 2015; Sadler et al., 2017). The initial position was seated with the trunk at a 90-degree angle to the thigh (Fig. 2).

#### Familiarisation protocol

Participants first attended two familiarisation sessions of 90 min on the FEMD and the procedures. The familiarisation consisted of a general warm up for both test sessions, consisting of five minutes of jogging (beats per minute <130; measured with a Polar M400), five minutes of joint mobility and three sets of 15s of frontal plank and glute bridge. The general warm-up was followed by four sets of five repetitions (two submaximal repetitions and three maximum repetitions) at a velocity of 0.15 m s^−1^ and 0.45 m s^−1^with a short range of movement (R_1_ = 25%) and a large range of movement (R_2_ = 50%). There was a three-minute pause between sets.

#### Test protocol

Participants arrived in a well-rested condition at the start of each testing session.

**Figure 3:**
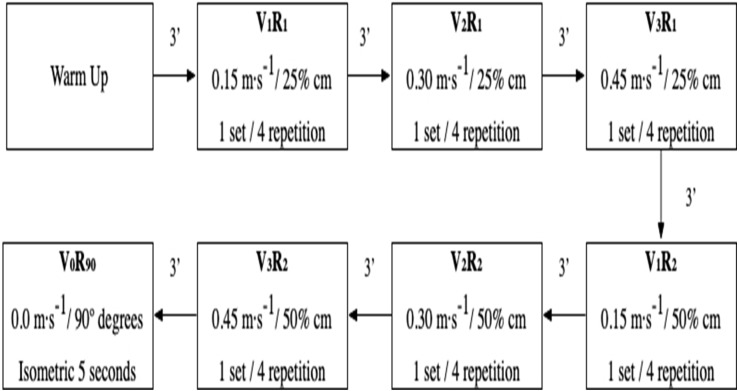
Exemplary experimental traces.

The instructions to the participants were always the same and no feedback was ever given to them. After the same warm-up as during the familiarisation protocols, participants rested for five minutes before the initiation. The test consisted of six series of four maximum consecutive repetitions, of trunk flexors at V_1_R_1_ (0.15 m s^−1^,25% cm), V_2_R_1_ (0.30 m s^−1^, 25% cm), V_3_R_1_ (0.45 m s^−1^, 25% cm), V_1_R_2_ (0.15 m s^−1^, 50% cm), V_2_R_2_ (0.30 m s^−1^, 50% cm), V_3_R_2_ (0.45 m s^−1^, 50% cm) and V_0_R_90_ (0 m s^−1^, 90 degrees). There was a three-minute pause between sets. After that, a maximum isometric contraction of five seconds, in a seated position with the trunk at a 90-degree angle to the thigh, was performed (Fig. 3).

### Statistical analysis

#### Outcome variables

The three highest repetitions of the mean force and the peak force for the concentric and the eccentric contractions were taken to calculate the dynamic force. In the calculation of the isometric force, the peak value and mean value of the repetition were taken.

#### Sample size

For test-retest evaluations without a control group, statistical theory predicts confidence intervals (d) = ±t0.975, n-1 ⋅ s ⋅$\sqrt{2}$/$\sqrt{n}$ for changes in the mean, where n is the sample size, s the SEM and t the statistician, equaling the expression *n* = 2(*ts*∕*d*)^2^ = 8s^2^/d^2^. The sample size is proportional to the square of the SEM. Previous studies (Dervisevic, Hadzic & Burger, 2007; Guilhem et al., 2014; Roth et al., 2017) have shown SEM values close to 8% with isokinetic parameters of trunk muscle strength. Assuming that the minimum clinically relevant change (d) be of 5%, then the sample size would be a minimum of 21 subjects.

#### Reliability

The descriptive data are presented as mean ±SD. The distribution of the data was verified by the Shapiro–Wilk normality test. Reliability was assessed by t-tests of paired samples with the effect size (ES), the CV and the ICC, with 95% confidence intervals. The scale used for interpreting the magnitude of the ES was specific to training research: negligible (<0.2), small (0.2–0.5), moderate (0.5–0.8), and large (≥0.8) (Cohen, 1988). The reliability observed in each evaluation condition was reported using the FEMD. For the relation between isometric tests and dynamic tests, a Pearson correlation coefficient was calculated with a 95% confidence interval. Following Hopkins et al. (2009), we classified the magnitude of the values of the intraclass correlation coefficient through a qualitative scale: values close to 0.1 are considered low reliability; 0.3, moderate; 0.5, high; 0.7, very high; and those close to 0.9, extremely high. Reliability analyses were performed using a customized spreadsheet (Hopkins, 2015), while JASP software package (version 0.9.1.0, http://www.jasp-stats.org) was used for all other analyses.

## Results

**Figure 4:**
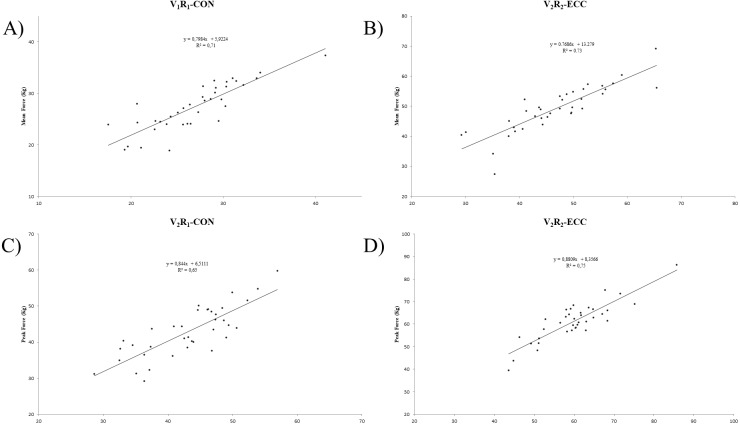
(A) Linear correlation of V1R1 condition in the concentric phase between test-retest of mean force of trunk flexors. (B) Linear correlation of V2R2 condition in the eccentric phase between test-retest of mean force of trunk flexors. (C) Linear correlation of V2R1 condition in the concentric phase between test-retest of peak force of trunk flexors. (D) Linear correlation of V2R2 condition in the eccentric phase between test-retest of peak force of trunk flexors.

The evaluation of the mean force of the concentric contraction of trunk flexors did not differ between the test and the retest (*p* > 0.05, ES < 0.20). However, the evaluation of the mean force of the eccentric contraction of trunk flexor strength was sensitive to the test and retest (*p* < 0.05, ES 0.13–0.32). Similarly, there was no significant difference between the test and retest of the peak force of concentric and eccentric contraction of trunk flexors (*p* > 0.05, ES < 0.20). The absolute reliability provided a stable repeatability for the isometric and dynamic protocols, with CV being below 10% in nearly all instances. The relative reliability of different strength protocols to evaluate the mean force of trunk flexors was very high (ICC = 0.71–0.85) for concentric contraction and very high (ICC = 0.74–0.86) and high (ICC = 0.69) for eccentric contraction (Table 2). Moreover, the absolute reliability provided better results in eccentric contraction (CV = 5.70–7.76) than concentric contraction (CV = 7.04–14.00). The relative reliability of different strength protocols to evaluate the peak force of trunk flexors was very high (ICC = 0.72–0.81) and high (ICC = 0.54–0.60) for concentric contraction and very high (ICC = 0.71–0.91) for eccentric contractions (Table 3). The most reliable conditions in which to evaluate flexor strength are presented in Fig. 4.

**Table 2:** Test–retest reliability of mean force measurements (Kg) provided by the functional electromechanical dynamometer at different velocities and ranges of movements.

	Conditions	Test	Retest	*p*-value	ES	CV (95% CI)	SEM (95% CI)	ICC (95% CI)
ISO	V_0_R_90°_	30.8 (5.4)	30.3 (5.4)	0.337	−0.11	8.21 (6.77–10.66)	2.51 (2.04–3.26)	0.79 (0.63–0.89)
CON	V_1_R_1_	26.9 (4.8)	27.4 (4.5)	0.251	0.11	6.82 (5.54–8.85)	1.85 (1.50–2.40)	0.85 (0.73–0.92)
	V_2_R_1_	26.0 (5.2)	26.6 (5.3)	0.328	0.10	9.06 (7.37–11.77)	2.38 (1.94–3.10)	0.80 (0.65–0.89)
	V_3_R_1_	24.8 (5.3)	25.1 (4.5)	0.537	0.08	10.83 (8.81–14.06)	2.70 (2.20–3.51)	0.71 (0.51–0.84)
	V_1_R_2_	23.7 (4.4)	24.3 (4.5)	0.200	0.13	8.00 (6.51–10.39)	1.92 (1.56–2.49)	0.82 (0.68–0.91)
	V_2_R_2_	23.6 (5.4)	24.1 (4.9)	0.337	0.11	10.18 (8.27–13.22)	2.43 (1.97–3.15)	0.79 (0.63–0.88)
	V_3_R_2_	22.9 (5.1)	23.9 (4.9)	0.096	0.18	9.89 (8.04–12.84)	2.31 (1.88–3.01)	0.79 (0.64–0.89)
ECC	V_1_R_1_	46.0 (9.0)	48.7 (7.6)	0.003	0.32	7.67 (6.24–9.96)	3.63 (2.95–4.72)	0.82 (0.68–0.90)
	V_2_R_1_	48.7 (8.7)	50.9 (8.0)	0.021	0.26	7.78 (6.33–10.11)	3.88 (3.15–5.04)	0.79 (0.63–0.89)
	V_3_R_1_	52.0 (9.1)	54.4 (7.7)	0.043	0.28	8.98 (7.30–11.66)	4.78 (3.88–6.21)	0.69 (0.47–0.83)
	V_1_R_2_	45.4 (7.7)	46.3 (7.2)	0.265	0.13	8.03 (6.53–10.43)	3.68 (2.99–4.78)	0.77 (0.59–0.87)
	V_2_R_2_	46.8 (8.5)	49.2 (7.7)	0.002	0.30	6.55 (5.33–8.51)	3.14 (2.56–4.08)	0.86 (0.74–0.92)
	V_3_R_2_	48.3 (9.7)	50.7 (9.1)	0.040	0.26	9.88 (8.04–12.84)	4.89 (3.98–6.35)	0.74 (0.55–0.86)

**Notes.**

ISO isometric contraction CON concentric contraction ECC eccentric contraction V_0_ 0 m s^−1^ V_1_ 0–15 m s^−1^ V_2_ 0–30 m s^−1^ V_3_ 0–45 m s^−1^ R_90_ 90 degrees R_1_ 25% cm R_2_ 50% cm ES Cohen’s d effect size CV coefficient of variation SEM standard error of measurement ICC intraclass correlation coefficient 95% CI 95% confidence interval

**Table 3:** Test–retest reliability of peak force measurements (Kg) provided by the functional electromechanical dynamometer at different velocities and ranges of movements.

	Conditions	Test	Retest	*p*-value	ES	CV (95% CI)	SEM (95% CI)	ICC (95% CI)
ISO	V_0_R_90°_	37.5 (6.7)	36.7 (5.6)	0.212	−0.14	7.72 (6.27–10.02)	2.86 (2.33–3.72)	0.79 (0.64–0.89)
CON	V_1_R_1_	42.2 (5.9)	42.6 (5.9)	0.721	−0.05	8.89 (7.23–11.55)	3.80 (3.09–4.94)	0.60 (0.34–0.77)
	V_2_R_1_	43.2 (6.7)	42.9 (7.0)	0.755	−0.03	7.04 (5.73–9.15)	3.03 (2.47–3.94)	0.81 (0.66–0.90)
	V_3_R_1_	44.8 (9.4)	46.2 (9.1)	0.344	0.15	14.00 (11.39–18.19)	6.37 (5.18–8.27)	0.54 (0.26–0.73)
	V_1_R_2_	36.9 (6.4)	37.1 (5.6)	0.780	0.04	8.79 (7.15–11.42)	3.26 (2.65–4.23)	0.72 (0.51–0.84)
	V_2_R_2_	37.7 (6.8)	38.1 (6.7)	0.573	0.06	8.35 (6.79–10.85)	3.17 (2.58–4.11)	0.79 (0.63–0.89)
	V_3_R_2_	40.2 (7.2)	40.4 (7.2)	0.898	0.02	11.40 (9.27–14.81)	4.60 (3.74–5.97)	0.60 (0.35–0.77)
ECC	V_1_R_1_	58.2 (9.1)	58.7 (7.9)	0.586	0.05	6.15 (5.00–7.99)	3.60 (2.92–4.67)	0.83 (0.69–0.91)
	V_2_R_1_	65.6 (7.5)	64.7 (8.8)	0.354	−0.11	6.41 (5.22–8.33)	4.18 (3.40–5.42)	0.75 (0.57–0.86)
	V_3_R1	73.7 (10.0)	74.5 (9.6)	0.525	0.08	7.31 (5.94–9.49)	5.42 (4.40–7.04)	0.71 (0.50–0.84)
	V_1_R_2_	57.1 (8.8)	57.3 (8.2)	0.823	0.02	6.93 (5.64–9.00)	3.97 (3.22–5.15)	0.79 (0.63–0.89)
	V_2_R_2_	60.0 (8.4)	61.2 (8.5)	0.101	0.14	5.07 (4.13–6.59)	3.08 (2.50–4.00)	0.87 (0.77–0.93)
	V_3_R_2_	66.8 (10.1)	66.3 (9.7)	0.665	−0.05	7.76 (6.31–10.08)	5.16 (4.20–6.71)	0.74 (0.55–0.86)

**Notes.**

ISO isometric contraction CON concentric contraction ECC eccentric contraction V_0_ 0 m s^−1^ V_1_ 0–15 m s^−1^ V_2_ 0–30 m s^−1^ V_3_ 0–45m s^−1^ R_90_ 90 degrees R_1_ 25% cm R_2_ 50% cm ES Cohen’s d effect size CV coefficient of variation SEM standard error of measurement ICC intraclass correlation coefficient 95% CI 95% confidence interval

The most reliable strength manifestation (CV = 6.82%) to evaluate the concentric contraction of trunk flexors was mean force with V_1_R_1_ condition; the most reliable strength manifestation to evaluate the eccentric contraction of trunk flexors was peak force with V_1_R_2_ (CV = 5.07%), and the most reliable strength manifestation to evaluate the isometric contraction of trunk flexors was peak force (CV = 7.72%) (Tables 2–3).

The peak force of concentric trunk flexor strength under V_1_R_1_ conditions was best related to the maximum isometric contraction (*r* = 0.70) and the mean force of eccentric trunk flexor strength with V_3_R_1_ conditions (*r* = 0.73; see Table 4).

**Table 4:** Correlation coefficient (r) from Pearson correlation analysis between isometric peak and mean force with dinamic peak and mean force (Kg).

Conditions		Mean force		Peak force	
		r	*p*-value	r	*p*-value
ISO-CON	V_1_R_1_	0.64	<.001	0.70	<.001
	V_2_R_1_	0.65	<.001	0.45	0.005
	V_3_R_1_	0.67	<.001	0.48	0.003
	V_1_R_2_	0.57	<.001	0.47	0.003
	V_2_R_2_	0.58	<.001	0.50	0.001
	V_3_R_2_	0.43	0.008	0.38	0.019
ISO-ECC	V_1_R_1_	0.58	<.001	0.59	<.001
	V_2_R_1_	0.61	<.001	0.52	0.001
	V_3_R_1_	0.73	<.001	0.48	0.003
	V_1_R_2_	0.48	0.003	0.58	<.001
	V_2_R_2_	0.56	<.001	0.54	<.001
	V_3_R_2_	0.55	<.001	0.50	0.002

**Notes.**

ISO isometric contraction CON concentric contraction ECC eccentric contraction V_1_ 0.15 m s^−1^ V_2_ 0.30 m s^−1^ V_3_ 0.45 m s^−1^ R_90_ 90 degrees R_1_ 25% cm R_2_ 50% cm

## Discussion

In the present study, we assessed the absolute and relative reliability of functional trunk tests using a FEMD (Dynasystem, Symotech). The reliability of strength test results is crucial to assess the level of adequate performance and develop a successful rehabilitation or training program (Demoulin et al., 2012). The main findings of the present study were very high, and high absolute and relative reliability was found in all assessment conditioning analyzed.

These findings are comparable to the results of Roth et al. (2017), who found a CV of 7.3% when evaluating isometric flexion trunk strength using a isokinetic device (IsoMed-200). In addition, flexion trunk strength was evaluated with the same device at 60° /s and 150° /s speed obtaining a CV of 7.8% and 18.4% respectively with a range of movement from−30° to 30° (Roth et al., 2017). Jubany et al. (2015) found similar results when using a custom-made instrument including a hand-held dynamometer for measuring isometric trunk flexor muscle strength, obtaining a CV of 5.3% and a CV of 6.6% when using the gold standard Back-Check (Jubany et al., 2015).

In the literature, many different ranges of movement and velocities are considered for studying the reliability of trunk flexor strength measurement using isokinetics devices (Dervisevic, Hadzic & Burger, 2007; Guilhem et al., 2014; Roth et al., 2017). However, no standardized protocol was established for obtaining higher reliability. Therefore, the current study compares the range of movement and the velocities to determine which one is more reliable. By analyzing the data obtained in Tables 2 and 3, it can be verified that the absolute and relative reliability is similar in all evaluation conditions. However, the condition that presents greater reliability in the concentric phase, whether we observe mean force or peak force values, is when it is evaluated at short ranges (CV = 6.82; CV = 7.04). This is not the case in the eccentric phase, where it is higher when evaluated at large ranges (CV = 6.55; CV = 5.07).

The data suggest that the concentric phase of the trunk flexors should be evaluated at a velocity of 0.15 m s^−1^ if we take mean force values, and at a velocity of 0.30 m s^−1^ if we take peak force values. The eccentric phase, whether we take mean force values or peak force values, should be evaluated at the velocity of 0.30 m s^−1^. Nevertheless, in the eccentric phase of the trunk flexors, there was a learning effect between the test and the retest when values of mean force were taken (*p* < 0.05). More familiarisation sessions would be needed to evaluate the eccentric phase in the trunk flexors. These results are consistent with other studies that have shown there is a learning effect in isokinetic devices (Keller, Hellesnes & Brox, 2001; Gruther et al., 2009). Perhaps there is a greater learning effect in the eccentric phase because in the day to day we do not perform this type of force as much as the concentric phase.

Additionally, we compare the absolute and relative reliability of the mean force and peak force of the trunk flexors. There are no major differences to help determine whether it is better to use mean force or peak force to evaluate trunk flexor strength with FEMD. In the clinical area, and sports performance, there is a tendency to use peak force values of trunk flexors for the study of reliability (Yasuda, Minami & Daikuya, 2013; Roth et al., 2017; Ben Moussa Zouita et al., 2018); however, there are no studies in which the differences between using peak force or mean force are described. Even so, the use of peak force can overestimate the force produced by the overshoot in the isokinetic devices (Delitto et al., 1991; Timm et al., 1992).

One of the innovations of this research was to study what evaluation condition is more related to isometric strength. The mean force of eccentric trunk flexor strength with V_3_R_1_ condition (*r* = 0.73) was best related to the maximum isometric contraction. There are no previous studies that analyse this, but it could be interesting to evaluate patients with low back pain because of the unrestricted range of movement in the sagittal plane (Balagué et al., 2010; Laird et al., 2014; Laird et al., 2019). These types of patients sometimes cannot make maximum dynamic efforts but they could make maximum isometric efforts. Thanks to knowing this relationship, the training loads in dynamic could be established.

The limitations of our study are as follows: first, we have only evaluated male students without any back pain, so our data can not be extrapolated to the rest of the population. Second, using a novel device and offering linear values of velocity and range of movement has made our research more difficult to compare with other studies and there was a learning effect with some effect sizes greater than 20% in the eccentric assessment. Future research could perform the same evaluation in another type of population, such as patients with low back pain, people of another age range and even female students, to further determine the best evaluation condition in these types of population.

The application of this test for the clinical or sports field allows us to know the muscular strength of the trunk flexors. With the data obtained in this study, we can understand what coefficient of variation the test has, and thereby know the effects of special training or rehabilitation programs. On the other hand, a reliable evaluation protocol with FEMD has been created, allowing us to know the best velocity and range of movement for evaluating this musculature. All of the protocols evaluated have shown reliable values, so that all of the evaluation conditions could be used. The advantage of using the FEMD is that it is cheaper than isokinetic devices and allows evaluating muscle strength in a more functional way without using so many straps.

This allows coaches and medical practitioners to match the sporting gesture to the evaluation. In the clinical field it is recommended to use the most reliable protocol; however, in the sports field, it is recommended to use the velocity and range closest to the sport performed.

## Conclusions

Based on the results of this study, it may be reasonably concluded that the assessment of the concentric, eccentric and isometric strength in the trunk flexors with FEMD is a reliable evaluation system. In this type of evaluation, data of mean force or peak force can be measured to determine the muscle strength without this influencing the reliability of the evaluation and if we want to relate the isometric strength with dynamic strength the V_3_R_1_ condition should be taken.

##  Supplemental Information

10.7717/peerj.7883/supp-1 Supplemental Information 1 Raw Data

## Additional Information and Declarations

### Data Availability

The following information was supplied regarding data availability: The raw data are available in the Supplemental Files. The raw data shows test and retest trunk flexors strength values of all conditioning.
